# Mobile Health Intervention Promoting Physical Activity in Adults Post Cardiac Rehabilitation: Pilot Randomized Controlled Trial

**DOI:** 10.2196/20468

**Published:** 2021-04-16

**Authors:** Linda G Park, Abdelaziz Elnaggar, Sei J Lee, Stephanie Merek, Thomas J Hoffmann, Julia Von Oppenfeld, Nerissa Ignacio, Mary A Whooley

**Affiliations:** 1 Department of Community Health Systems School of Nursing University of California San Francisco San Francisco, CA United States; 2 San Francisco Department of Veterans Affairs Health Care System San Francisco, CA United States; 3 Division of Geriatrics School of Medicine University of California San Francisco San Francisco, CA United States; 4 Institute for Human Genetics University of California San Francisco San Francisco, CA United States; 5 Departments of Medicine and Epidemiology & Biostatistics University of California San Francisco San Francisco, CA United States

**Keywords:** physical activity, cardiac rehabilitation, digital health, mobile app, wearable device, mHealth

## Abstract

**Background:**

Cardiac rehabilitation (CR) is an exercise-based program prescribed after cardiac events associated with improved physical, mental, and social functioning; however, many patients return to a sedentary lifestyle leading to deteriorating functional capacity after discharge from CR. Physical activity (PA) is critical to avoid recurrence of cardiac events and mortality and maintain functional capacity. Leveraging mobile health (mHealth) strategies to increase adherence to PA is a promising approach. Based on the social cognitive theory, we sought to determine whether mHealth strategies (Movn mobile app for self-monitoring, supportive push-through messages, and wearable activity tracker) would improve PA and functional capacity over 2 months.

**Objective:**

The objectives of this pilot randomized controlled trial were to examine preliminary effects of an mHealth intervention on group differences in PA and functional capacity and group differences in depression and self-efficacy to maintain exercise after CR.

**Methods:**

During the final week of outpatient CR, patients were randomized 1:1 to the intervention group or usual care. The intervention group downloaded the Movn mobile app, received supportive push-through messages on motivation and educational messages related to cardiovascular disease (CVD) management 3 times per week, and wore a Charge 2 (Fitbit Inc) activity tracker to track step counts. Participants in the usual care group wore a pedometer and recorded their daily steps in a diary. Data from the 6-minute walk test (6MWT) and self-reported questionnaires were collected at baseline and 2 months.

**Results:**

We recruited 60 patients from 2 CR sites at a community hospital in Northern California. The mean age was 68.0 (SD 9.3) years, and 23% (14/60) were female; retention rate was 85% (51/60). Our results from 51 patients who completed follow-up showed the intervention group had a statistically significant higher mean daily step count compared with the control (8860 vs 6633; *P*=.02). There was no difference between groups for the 6MWT, depression, or self-efficacy to maintain exercise.

**Conclusions:**

This intervention addresses a major public health initiative to examine the potential for mobile health strategies to promote PA in patients with CVD. Our technology-based pilot mHealth intervention provides promising results on a pragmatic and contemporary approach to promote PA by increasing daily step counts after completing CR.

**Trial Registration:**

ClinicalTrials.gov NCT03446313; https://clinicaltrials.gov/ct2/show/NCT03446313

## Introduction

### Background

Cardiovascular disease (CVD) is the leading cause of mortality, affecting 43.7 million older adults aged 60 years and over [1]. After a cardiac event such as myocardial infarction, coronary revascularization, or valve procedure, Class 1A national performance measures recommend that patients be referred to cardiac rehabilitation (CR) [2-4]. CR programs, which consist of supervised exercise training, behavioral activation, and psychosocial support [4,5], promote physical activity (PA) and other health behaviors that reduce secondary events and mortality [6]. Upon graduation from a typically 12-week CR program, patients are encouraged to continue these same levels of physical activity (PA) indefinitely [7]. However, numerous studies have shown that most patients fail to maintain the recommended levels of PA and instead return to a sedentary lifestyle [8].

Maintaining PA after CR is particularly important in older adults to gain and maintain the critical benefits of improved physical function (balance, gait, strength, and endurance) [9]. PA maintenance after CR is linked to reduced adverse geriatric outcomes such as falls and mobility impairment [9], thereby increasing susceptibility to adverse secondary cardiac events, functional decline, and depression [5]. Compared with other groups, older adults benefit from extended education on safety of exercising independently and motivation to maintain PA. Thus, more targeted interventions for older adults on promoting PA maintenance after CR completion are clearly needed.

As smartphone ownership increases across age groups worldwide [10], mobile technology has become more integrated with health care, and use of mobile health (mHealth) apps has become feasible for even the most novice users [11]. Health apps can be effective adjuncts for the management of chronic conditions [12] and have a positive impact on long-term behavior change [13]. Therefore, we sought to determine whether an mHealth intervention with a wearable activity tracker, mobile app, and text messages could promote PA maintenance after CR completion. The aims of the Mobile4Heart pilot randomized controlled trial (RCT) were to examine preliminary effects of an mHealth intervention on group differences in PA (step counts) and functional capacity measured by the 6-minute walk test (6MWT) and group differences in depression and self-efficacy to maintain exercise after CR. We hypothesized that the intervention group would have higher levels of PA, greater functional capacity, less depression, and higher self-efficacy compared with the control group after 2 months of using supportive technology to maintain PA.

### Theoretical Framework

Behavior change in PA has been more successful with theory-based interventions [14] such as social cognitive theory (SCT) [15,16]. We applied the tenets of SCT to develop the intervention components and explain the mechanism of behavior change in PA maintenance after completing CR. SCT is one of the most commonly used behavioral change theories in PA research [17,18] and posits that human behavior is a triadic and reciprocal interaction of one’s personal (cognitive and affective), behavioral (actions and reactions), and environmental (social and physical) factors [19]. Our intervention components include the 3 major SCT constructs: (1) self-efficacy (one’s perception of their ability to perform a particular behavior); (2) self-regulation (ability to exert control over their behavior, cognitions, and environment); and (3) social support (emotional, instrumental, or informational help from a social network). SCT represents a causal model in which self-efficacy affects human behavior through other mediating processes such as mastery of self-regulation and building social support [20-23]. In the context of our intervention (Table 1), using the proposed mHealth technology is closely related to SCT constructs as it is proposed to build self-efficacy, self-regulation skills, and perceived social support, thus leading to PA maintenance.

**Table 1:** Social cognitive theory components.

Social cognitive theory construct	mHealth intervention	Control
Self-efficacy	Mobile app (Movn)	Paper-and-pencil diary
Self-regulation	Fitbit Charge 2	Pedometer
Social support	2 telephone calls plus tailored motivational text messages	2 telephone calls

## Methods

### Ethical Approval

This study was approved by the institutional review boards at the John Muir Medical Center (recruiting) and University of California San Francisco (sponsoring).

### Study Sample and Participants

**Figure 1:**
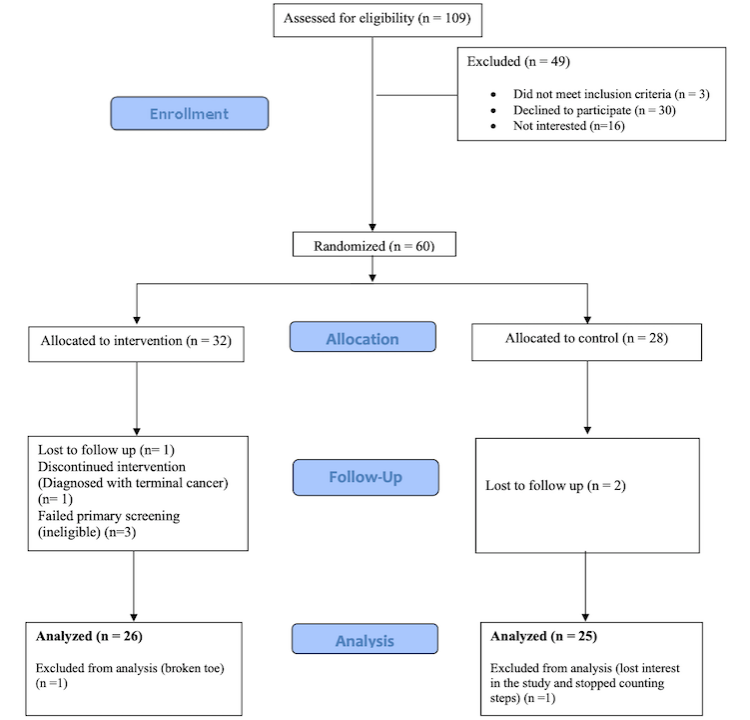
Consolidated Standards of Reporting Trials screening and recruitment diagram.

Between February 2018 and January 2019, 109 patients at 3 community CR centers were screened for eligibility. A total of 60 participants were included and randomized (using computer-based randomization) to the intervention group or usual care (control) group (Figure 1). Participants were included in the study if they were at least age 18 years, had a history of CVD, owned a smartphone or tablet, and were within 2 weeks of completing CR. Exclusion criteria included cognitive impairment, lack of English proficiency or literacy, or unstable clinical conditions (eg, unstable arrhythmias, uncontrolled hypertension, active infection, second or third degree heart block).

### Study Design and Intervention

We conducted a pilot RCT with 2 arms. The intervention group received (1) a Charge 2 (Fitbit Inc) to record step counts, (2) a Movn mobile app to record exercise, and (3) push-through motivational PA prompts and educational messages related to CVD management. Messages were sent from the study team through the Movn app as a push notification 3 times per week on random days between 9 AM and 6 PM to provide positive feedback and additional motivation for PA. These 1- and 2-way messages were based on the American Heart Association Simple 7 principles [24] and prompted participants to engage in PA, keep healthy eating habits, or track their medication use.

Additionally, participants could use the Movn app to record daily weight, blood pressure, heart rate, medication use, and other exercise (eg, swimming, biking) not captured by the Fitbit device. Every time they chose to record any of these other measures, they were prompted to complete this information through a push notification from the app. Finally, mHealth participants had the ability to report any cardiovascular symptoms through the Movn app. If the participant reported shortness of breath or chest pain, a message prompted them with a button to call 911. The study team triaged participant entries once a day.

Participants in the control group were provided a basic pedometer (Walking 3D, IceFox) and paper-and-pencil diary to record daily step counts. They were asked to fasten the pedometer around their waist or to place it in their pants pocket during all waking hours.

All participants received a phone call (or text message if in the intervention group) 3 days following enrollment to answer any questions regarding the study and verify adherence to the assigned regimen. After 1 month of participation, participants received a follow-up phone call or text sent by the study staff.

### Procedures

Participants in the intervention group wore the Charge 2 on their wrist during all waking hours. Fitbit devices use a 3-axis accelerometer and translate movement into digital measurements when attached to the body providing information about the frequency, duration, intensity, and patterns of movement to determine the number of steps taken, distance traveled, calories burned, and sleep quality [25]. For the purpose of this study, only the number of steps was collected.

Participants from the intervention group downloaded the Movn rehab mobile app and Fitbit app to their smartphone, the latter being used to wirelessly sync and view step counts. To protect patients’ information, a generic email account and initials were generated by the study staff to register the participants on both Movn and Fitbit apps. The study team provided technical support to participants as needed.

### Outcome Measures and Data Collection

Two study staff met with all enrolled participants for the baseline visit within 2 weeks of completing CR. A standardized script was used as a checklist to ensure all components of enrollment were covered. All participants provided written informed consent before participation. Cognitive assessment was conducted for each participant using the Mini-Cog test [26-28]. Participants scoring positive for cognitive impairment on the Mini-Cog test were considered a screen failure. Once eligibility was determined, participants completed paper questionnaires about sociodemographic characteristics, mobile phone use, exercise self-efficacy, quality of life, self-reported PA, and depression. Participants then completed the 6MWT. A 60-meter corridor in the CR building was designated in which to perform the 6MWT. The wall was premarked with 30 meters for the one length of the corridor and patients were asked to walk back and forth as many times as possible over 6 minutes. Two cones were placed at both ends of the long corridor. Patients were allowed to take breaks if needed while the timer kept going. Vital signs were collected just before and after the test including blood pressure, heart rate, and oxygen saturation. Upon completion of the baseline questionnaires and 6MWT, patients were informed as to which study group they were randomized.

Upon completion of the 2-month study period, participants returned for a follow-up visit. At this visit, participants completed the following questionnaires about exercise self-efficacy, quality of life, self-reported PA, and depression. All participants performed the 6MWT. For participants in the control group, they also submitted their step diary. As compensation for time spent in the study, participants in the intervention group chose between keeping their Charge 2 or receiving $100 in the form of gift cards. Participants in the control group received $100 in gift cards.

This pilot study assessed the feasibility of supporting older adults using an mHealth intervention through successes in screening, recruitment, and retention. Acceptability was measured using a satisfaction questionnaire. In addition, we conducted individual interviews with 7 participants from the intervention group to obtain additional feedback that will be reported in a future publication.

The outcome of step counts was collected from the intervention group using Fitabase [29]. Fitabase is a comprehensive data management platform, stored in a cloud format, designed to support all data collection from the Fitbit device [29]. Data were automatically collected once the Fitbit activity trackers were synced with the Fitbit app. For the control group, steps data were collected from the diaries provided to the participants during study enrollment.

To determine functional capacity, 6MWT data were collected at baseline and 2-month follow-up for all participants [30-34]. Self-report data measuring psychosocial variables and PA were collected at baseline and follow-up.

We assessed participants for change in depressive symptoms and exercise self-efficacy from baseline to 2 months. The Patient Health Questionnaire (PHQ)–9 is a self-report instrument for depressive symptoms (Cronbach α=0.88); higher scores on the PHQ-9 (range 0 to 27) correspond with greater depressive symptom severity [35,36]. The Exercise Self-Efficacy Scale (EXSE) was used to determine participant confidence in their ability to exercise in the future; higher scores (range 0% to 100%) indicate higher self-confidence to exercise (Cronbach α=0.92). We also examined these variables as covariates related to PA and functional capacity.

### Statistical Analysis

Descriptive statistics and outcomes are presented as means with standard deviations for continuous variables (or medians and interquartile ranges [IQR] if skewed) and proportions (%) for categorical variables. *P* values for baseline tests were calculated from 2-sample *t* tests (or Wilcox rank-sum test if skewed) or chi-square tests.

We analyzed the primary outcome of step count difference between the two groups over 60 days fitting linear mixed-effects models with the step count for each day [37] in R version 3.6.0 (The R Foundation for Statistical Computing) [38]. For a more precise estimate of the treatment effect, we used a stepwise procedure to include potential covariates impacting the outcome (age, gender, working status, college or higher education, relationship status, depression, self-efficacy, and self-reported PA), including only terms that were significant in the model. The 2-sided significance level was established a priori at an alpha of .05. We employed an intention-to-treat analysis approach.

We tested the functional capacity outcome of the standardized change score in 6MWT from baseline and 2 months also using linear mixed models, with the same approach. The covariate association of depression and self-efficacy for step count difference and 6MWT were also considered to be secondary questions of interest.

Since our analysis included a small amount of missing data for the covariates (education missing on 8%, all other covariates <4%), our analyses used multiple imputation by chained equations, using 5 imputations [39]. Results differed very little compared with complete case analysis.

## Results

### Characteristics of Study Participants

Figure 1 displays the screening and recruitment results of 60 participants according to the CONSORT (Consolidated Standards of Reporting Trials) guidelines. Table 2 outlines the characteristics of our sample that included 51 participants for the final analysis. The two groups did not differ significantly at baseline on any of the sociodemographic characteristics. Participants were aged 49 to 89 (mean 68.0) years. The majority were male (46/60, 77%) and identified as white (45/60, 75%). In addition, there were no group differences in self-reported baseline PA (high defined as exercising 150 minutes or more per week). Primary diagnoses for enrollment in CR included ischemic heart disease (eg, percutaneous coronary intervention, coronary artery bypass surgery, angina, and myocardial infarction; 49/60 [82%]), valvular heart disease (eg, aortic valve replacement, mitral valve replacement, etc, 6/60 [10%]), heart failure (3/60, 5%), and structural heart disease (eg, myxoma, aortic dissection, 2/60, 3%).

**Table 2:** Baseline sociodemographic data of enrolled participants (n=51).

Characteristic			Intervention (n=26)		Control (n=25)	*P* value
Age, mean (SD)			66.7 (8.6)		66.8 (8.7)	.97
Female, n (%)			6 (23)		5 (20)	.79
Hispanic or Latino, n (%)			1 (4)		2 (8)	.70
White race, n (%)			19 (73)		22 (88)	.37
Married, n (%)			23 (88)		19 (76)	.19
Employed, n (%)			10 (38)		13 (52)	.33
College graduate, n (%)			18 (69)		16 (64)	.92
Physically active, n (%)			20 (63)		16 (57)	.50
PHQ-9^a^, median (IQR)^b^			1 (0, 3)		2 (0, 3)	.53
EXSE^c^, median (IQR)			10 (9.9, 10)		9.9 (8.4, 10)	.07
6MWT^d^ (meters), mean (SE)			430 (112)		429 (97)	.96
Causes for enrollment in cardiac rehabilitation, n (%)						
	Ischemic heart diseases	19 (73)		22 (88)		.19
	Heart failure	4 (15)		1 (4)		.18
	Valvular heart disease	2 (8)		1 (4)		.58
	Structural heart disease	1 (4)		1 (4)		.98

^a^PHQ-9: Physical Health Questionnaire for depression.

^b^IQR: Interquartile range.

^c^EXSE: Exercise Self-Efficacy Scale.

^d^6MWT: 6-minute walk test.

### Feasibility and Acceptability

We screened an average of 3 individuals per week and were successful in recruiting 60 individuals over 11 months. We enrolled 60 individuals; however, 9 withdrew or were lost to follow-up (4 from the intervention group and 5 from the control group), representing 15% (9/60) attrition. We also measured overall acceptability by administering a satisfaction questionnaire at the end of study with overall high satisfaction scores for the Movn app and Fitbit device (4.5 and 4.86 out of 5, respectively) but lower scores for the push messages (3.14 out of 5).

### Physical Activity Outcome: Mean Daily Step Counts

**Figure 2:**
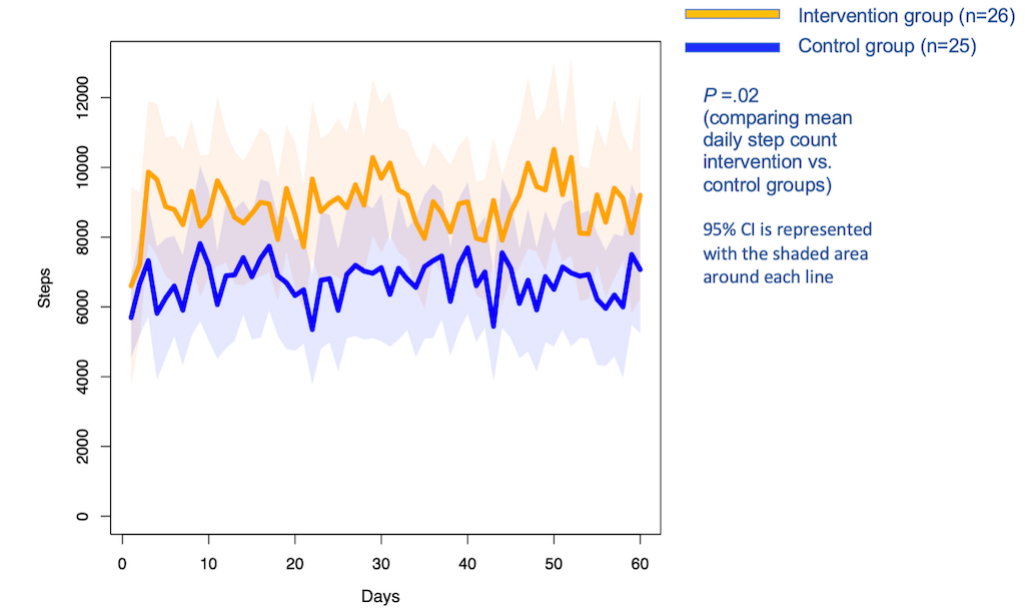
Mean daily step counts over 60 days.

Over the 2-month period, the intervention group showed a statistically significant higher mean daily step count compared with the control group (8860 vs 6633, respectively; or a covariate-adjusted difference of 2192 steps (95% CI 344 to 4040 steps, *P*=.02; Figure 2). This result was adjusted for age only, as the other covariates (race, working status, college education, depression, and exercise self-efficacy) were not significantly different when included in both univariate and multivariate linear models. The unadjusted difference in mean step counts was similar (difference of 2223 steps, 95% CI 138 to 4308 steps, *P*=.04). We also tested for any difference over time but found no significant change over the 2-month period (β=3.7 steps per day, 95% CI –3.1 to 10.6, *P*=.29).

### Functional Capacity Outcome

**Figure 3:**
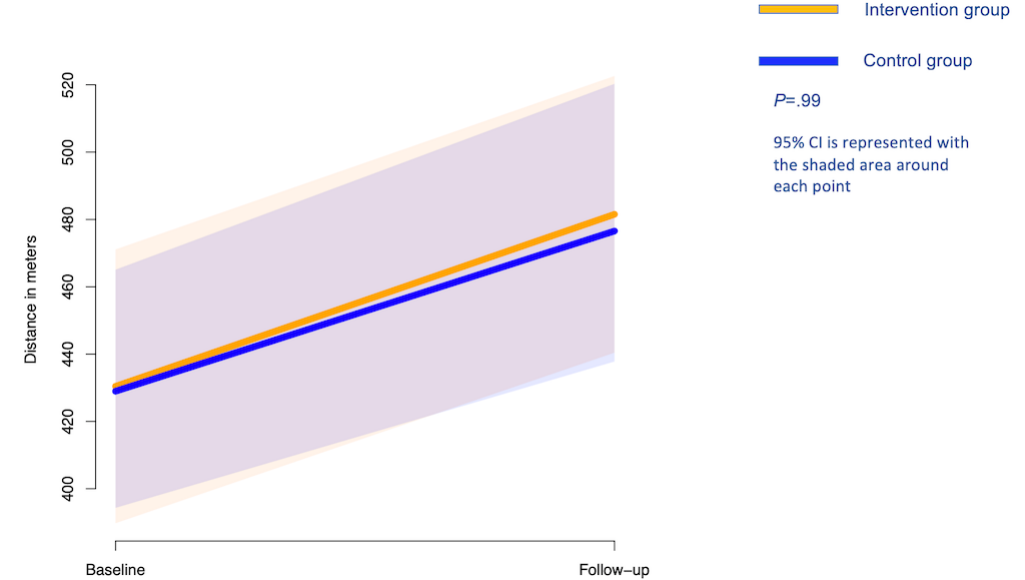
Change in exercise capacity over 60 days.

There were no statistically significant differences between the groups at follow-up in the other outcome measures evaluated (Figure 3). After 60 days of follow-up, mean 6MWT distance increased by 61 meters in both intervention and control groups, but there was no significant difference between the two groups (0.4 meters, 95% CI 44.7 to 45.4 meters, *P*=.99). In this analysis, we adjusted for age, gender, and college or higher education in our final multivariate model. However, there was a main effect of time (an increase of 138 steps from baseline to after 60 days of follow-up (95% CI 77 to 199), or 2.3 steps per day, (95% CI 1.3 to 3.3), *P*=<.001), after adjusting for the same covariates.

### Depressive Symptoms and Exercise Self-Efficacy Outcomes

Total scores for depressive symptoms and exercise self-efficacy were examined from baseline to 2 months. We also examined these variables as possible covariates related to the PA and 6MWT results. There was no significant association between depressive symptoms and exercise self-efficacy with step counts or distance walked in the 6MWT for either group in univariate or multivariate analyses (Table 3).

**Table 3:** Association of depression (Physical Health Questionnaire for depression) and exercise self-efficacy (Exercise Self-Efficacy Scale) with step count and 6-minute walk test.

Covariate	Intervention	Control	β_Steps_^a^ (95% CI)	*P*_Steps_^b^	β_6MWT_^c,d^ (95% CI)	*P*_6MWT_^e^
PHQ-9^a,f^, n (%)	27.0 (90.0)	24.0 (85.7)	2390 (172, 4952)	.07	116 (–60, 292)	.21
EXSE^b,g^, n (%)	7.0 (25.9)	14.0 (50.0)	–1051 (–2998, 895)	.29	50 (–43, 142)	.30

^a^Score <5 meaning no depressive symptoms.

^b^Score <10 meaning lower than maximum exercise self-efficacy.

^c^6MWT: 6-minute walk test.

^d^Adjusted for age and group assignment.

^e^Adjusted for age, group assignment, and baseline versus follow-up, gender, and education.

^f^PHQ-9: Physical Health Questionnaire for depression.

^g^EXSE: Exercise Self-Efficacy Scale.

## Discussion

### Principal Findings

Maintenance of exercise during the critical time period immediately after discharge from CR has been shown to predict future health behaviors and outcomes [24]. Our pilot study provides a contribution to the literature by examining the outcomes of an mHealth intervention that incorporates a mobile phone app. The intervention included a mobile phone app to deliver push-through messages and notifications plus an activity tracker to maintain PA in a vulnerable, older patient population who experienced an adverse cardiac event requiring CR. This mHealth intervention that deployed multiple technologies was deemed feasible with a high retention rate in both study groups (85%) and attested to participants’ motivation to remain active and apply the knowledge they learned during CR. Participants also reported high satisfaction with the technology used in this study, including reminders to walk at least 250 steps every hour in times of inactivity.

Preliminary effects of the intervention were promising, with higher average daily step counts for the intervention group over 2 months of follow-up than those assigned to a control condition (pedometer + paper-and-pencil diary). Participants in the intervention group walked, on average, 2192 more daily steps than the control group. Our other outcomes of functional capacity, self-reported depressive symptoms, and exercise self-efficacy were not significantly different between groups. However, this study highlights the potential benefits of using a mobile app that was specifically designed for CR and a wearable device to promote PA after CR discharge among adults with CVD who had a mean age of 66.7 (SD 8.6) years in the intervention group. These promising data suggest that patients who participate in CR may benefit from the use of mobile technology after CR discharge to maintain PA, which is strongly associated with improved clinical outcomes such as less morbidity and mortality [40].

For optimal health cardiovascular outcomes, the American Heart Association and World Health Organization recommend 150 minutes of moderate-intensity aerobic PA per day for 5 days per week [41], which is about 7000 to 8000 steps per day. Studies suggest that 7500 steps per day are recommended for secondary prevention in patients with coronary artery disease to improve lipid profiles, muscle endurance, BMI, and waist circumference [42,43]. Other studies show that compared with <6000 steps per day, older adults with 8000 to 10,000 steps per day (equivalent to 20 to 30 minutes per day at an intensity >3 metabolic equivalents) have improved cardiovascular and musculoskeletal function [44]. Although this general recommendation may be a good starting point to improve PA for the target population in this study, the dose-response relationship between PA and health status may not be sufficient to reach optimal health status after a cardiac event or account for those with conditions such as heart failure or arthritis that may limit mobility. Moreover, there is no agreement between experts about the exact number of steps needed per day for tertiary prevention in older patients with CVD, and thus more research is needed for this at-risk population [45,46].

### Limitations

While this study showed promising PA outcomes for adults after CR, there are important limitations to consider. First, the accuracy of most commercially available activity tracking devices is unclear although studies found high positive correlation and agreement between Fitbit devices and the Actigraph accelerometer as well as pedometers and the Actigraph device [47-50]. Accuracy of step counts from most PA devices is based on gait patterns collected from healthy volunteers [51,52]. Second, this study used different activity tracking devices for the intervention group (Fitbit wrist band) and control group (waist-level or pocket pedometer). As the primary intervention was a wearable activity tracker, providing the control group with Fitbit devices would have imposed the risk of diluting the intervention effect. Studies have shown that positioning of the tracker on the body could alter the sensitivity of the device and subsequently influence the number of captured steps [53,54]. Third, this pilot study included a small sample size with a 2-month duration, not allowing for conclusive results that are fully powered or long-term results on PA maintenance after CR. Fourth, the final sample was lacking in racial and gender diversity with a majority of white, male participants who tend to have higher PA than other racial groups [55,56], which limits the generalizability of this study results. Fifth, it is possible the control group showed a change in behavior by walking more steps than their baseline due to wearing a pedometer that provided a form of feedback about their PA [57,58]. Last, we proposed the chosen mHealth technology (Movn app, Charge 2, text messages) supported the building of theory-based constructs of self-efficacy and self-regulation from SCT that would promote behavior change. However, we acknowledge that the use of a pedometer may have supported self-regulation, although we hypothesized to a lesser degree due to the limited interaction with the pedometer as compared with the Fitbit device.

### Comparison With Prior Work

Previous studies have reported that participants who used tracking tools to self-monitor PA reported significantly increased long-term adherence to regular exercise, which translated into better overall quality of life and reduction in risk factors [59-62]. Therefore, there have been several approaches to achieve this level of self-monitoring. To date, there have been few RCTs examining multiple technology-based interventions to sustain PA after completing CR (eg, text messages, online classes, and online social support groups) among the older adult population [63]. Many of these interventions were not tailored to participants’ individual goals and needs but instead involved general messaging and feedback. Key factors associated with successful interventions include personalized messages with tailored advice, high engagement (2-way text messaging, higher frequency of messages), and use of multiple modalities [64]. Interventions that do not included tailoring could lead to loss of motivation and high attrition. PA interventions should focus on active engagement of participants through tailored physical fitness goals, tracking their performance [63,65,66], and 2-way communication about their progress.

### Conclusion

Participants of CR receive little to no support during the transition from CR to community/home-based PA and need an organized support mechanism to maintain PA [39]. This pilot study showed an mHealth intervention using a wearable device and mobile phone app can increase PA with daily step counts in patients who complete CR. This intervention presented a pragmatic and contemporary approach for adults to promote PA after completing CR. This study provides support for a full-scale RCT with a longer intervention and monitoring period to assess trends in PA after CR as a result of applying mHealth technology for self-monitoring after CR. Future research will implement more tailored coaching for older adults. Our findings provide evidence for using mHealth to enhance patient self-management and demonstrate strong potential to promote PA maintenance through education, recording goals, tracking PA, and receiving tailored feedback.

## Appendix

Multimedia Appendix 1 CONSORT-EHEALTH checklist (V 1.6.1).

## Abbreviations

6MWT: 6 minute walk test
CONSORT: Consolidated Standards of Reporting Trials
CR: cardiac rehabilitation
CVD: cardiovascular disease
EXSE: Exercise Self-Efficacy Scale
IQR: interquartile range
mHealth: mobile health
PA: physical activity
PHQ-9: Patient Health Questionnaire
RCT: randomized controlled trial
SCT: social cognitive theory
